# A genetic analysis of *Trichuris trichiura* and *Trichuris suis* from Ecuador

**DOI:** 10.1186/s13071-015-0782-9

**Published:** 2015-03-19

**Authors:** Hayley Meekums, Mohamed BF Hawash, Alexandra M Sparks, Yisela Oviedo, Carlos Sandoval, Martha E Chico, J Russell Stothard, Philip J Cooper, Peter Nejsum, Martha Betson

**Affiliations:** Department of Production and Population Health, Royal Veterinary College, Hawkshead Lane, Hatfield, Herts AL9 7TA UK; Department of Veterinary Disease Biology, Faculty of Health and Medical Sciences, University of Copenhagen, Dyrlaegevej 100, Frederiksberg C, DK-1870 Denmark; Zoology Department, Faculty of Science, Cairo University, Giza, 12613 Egypt; Department of Parasitology, Liverpool School of Tropical Medicine, Pembroke Place, Liverpool, L3 5QA UK; Institute of Immunology and Infection Research, Centre for Immunity, Infection and Evolution, School of Biological Sciences, University of Edinburgh, King’s Buildings, Ashworth Laboratories, Charlotte Auerbach Road, Edinburgh, EH9 3FL UK; Laboratorio de Investigaciones FEPIS, Quinindé, Esmeraldas Province Ecuador; Centro de Investigaciónen Enfermedades Infecciosas, Pontificia Universidad Católica del Ecuador, Quito, Ecuador; Institute of Infection and Immunity, St George’s University of London, Cranmer Terrace, London, SW17 0RE UK

**Keywords:** *Trichuris*, Whipworm, Human, Pig, Ecuador, Zoonosis, Phylogenetics

## Abstract

**Background:**

Since the nematodes *Trichuris trichiura* and *T. suis* are morphologically indistinguishable, genetic analysis is required to assess epidemiological cross-over between people and pigs. This study aimed to clarify the transmission biology of trichuriasis in Ecuador.

**Findings:**

Adult *Trichuris* worms were collected during a parasitological survey of 132 people and 46 pigs in Esmeraldas Province, Ecuador. Morphometric analysis of 49 pig worms and 64 human worms revealed significant variation. In discriminant analysis morphometric characteristics correctly classified male worms according to host species. In PCR-RFLP analysis of the ribosomal Internal Transcribed Spacer (ITS-2) and 18S DNA (59 pig worms and 82 human worms), nearly all *Trichuris* exhibited expected restriction patterns. However, two pig-derived worms showed a “heterozygous-type” ITS-2 pattern, with one also having a “heterozygous-type” 18S pattern. Phylogenetic analysis of the mitochondrial large ribosomal subunit partitioned worms by host species. Notably, some Ecuadorian *T. suis* clustered with porcine *Trichuris* from USA and Denmark and some with Chinese *T. suis.*

**Conclusion:**

This is the first study in Latin America to genetically analyse *Trichuris* parasites. Although *T. trichiura* does not appear to be zoonotic in Ecuador, there is evidence of genetic exchange between *T. trichiura* and *T. suis* warranting more detailed genetic sampling.

## Findings

### Background

The soil-transmitted helminth (STH) *Trichuris trichiura* infects around 465 million people worldwide [1], being especially prevalent where hygiene and sanitation are poor. The closely-related *T. suis* infects innumerable pigs globally and is associated with significant economic losses [2]. As eggs, larvae and adults of *T. trichiura* and *T. suis* are morphologically indistinguishable, the extent of natural cross-transmission of *Trichuris* between humans and pigs is not known. Experimental infection studies reveal that *T. trichiura* can establish in pigs, although adult worms rarely persist, while patent *T. suis* infection has been observed in man [3].

The introduction of molecular techniques has provided insights into genetic differences between *T. trichiura* and *T. suis* and the potential for cross-transmission between host species. Analysis of mitochondrial and nuclear sequences in pig-derived and human-derived *Trichuris* has supported the proposition that *T. trichiura* and *T. suis* are distinct species [4,5]. However, transmission from pigs to humans in Uganda has been noted [6]. The transmission biology of trichuriasis in other parts of the world, e.g. Latin America, awaits investigation. In Ecuador, the estimated prevalence of *T. trichiura* infection is 9% [7], but prevalences are much higher in certain rural areas [8,9]. Epidemiological surveillance of *T. suis* in pigs is scant, but given that there are over 1.2 million pigs in the country [10] and backyard production systems are common, *T. suis* infections are likely to be widespread.

The aims of this study were to perform a genetic analysis of *Trichuris* collected from pigs and people in rural Ecuador to clarify the epidemiology of trichuriasis.

## Methods

A parasitological survey of humans and pigs was conducted in Quinidé and Súa Districts, Esmeraldas Province, Ecuador [11]. 32 families (n = 132) were enrolled. Pigs (n = 46) were selected for sampling based on their proximity to *Trichuris-*positive children. Participants were asked to provide a faecal sample and faecal samples were collected from pigs. Parasitological diagnosis was conducted by examination of duplicate Kato-Katz smears [12]. Ethical approval was provided by the Ethical Committees of Liverpool School of Tropical Medicine and Pontificia Universidad Catolica del Ecuador [11]. Written informed consent was provided by all participants or their guardians (for children). STH-positive individuals were treated with 400 mg albendazole or pyrantel pamoate (see below).

To collect adult worms, participants with *T. trichiura* counts ≥480 eggs per gram faeces were given pyrantel pamoate at 10 mg/kg over three days. Participants and their guardians were instructed to collect stools produced. Recovered worms were washed thoroughly before storage in 70% ethanol. *Trichuris-*positive pigs were treated with piperazine at 0.2 g/kg. Expelled worms were collected over the following two days. Morphometric analysis of adult *Trichuris* was carried out as described [6]. Discriminant analysis was conducted in SPSSv20 starting with four morphometric characteristics (female worms), which were removed or exchanged in a stepwise fashion to identify the most discriminatory combination. A maximum of three characteristics was used for male worms due to the small number obtained from pigs. The data for female worms did not meet the assumption of equal variance among groups.

Genomic DNA was extracted from *Trichuris* using the Wizard Genomic DNA Purification Kit. Internal Transcribed Spacer-2 (ITS-2) PCR-RFLP was conducted as described [6]. An 18S DNA PCR-RFLP was designed. Restriction sites were identified using Webcutter2.0 and *Alu*1 differentiated between human and pig *Trichuris*. The primers were: Tri18S_F1 (5′- CGAACGAGACTCTGGCCTAC) and Tri18S_R (5′- CCTTGTTACGACTTTTACTTCCTC). Cycling conditions were: denaturation at 95°C for 15 min, followed by 35 cycles of 95°C for 30s, 55°C for 1 min and 72°C for 1 min, with a final extension of 72°C for 5 min. PCR products (4 μl) were digested with 4U *Alu*1.

29 worms (15 from four humans and 14 from a pen containing four pigs) were chosen for mitochondrial large ribosomal subunit (*rrn*L) sequencing. 422 bp of *rrn*L were amplified using primers TrirrnLF (5′-TGTAAWTCTCCTGCCCAATGA) and TrirrnLR (5′-CGGTTTAAACTCAAATCACGTA). PCR conditions were: initial denaturation at 95°C for 15 min followed by 35 cycles of 95°C for 30s, 50°C for 30s and 72°C for 1 min and a final extension at 72°C for 10 min. For comparison, 10 pig worms from Denmark and USA were analysed and additional sequences were retrieved from GenBank: Chinese *T. suis* (AM993027-AM993032, HQ183734-HQ183736 and GU070737) and *T. trichiura* (AM993017-AM993022). Phylogenetic analysis using neighbor-joining and maximum-likelihood trees was conducted in MEGA6.1 [13] using jModelTest to identify the best model [14]. *Trichinella spiralis* (AF293969) was used as an outgroup. Ecuadorian *rrn*L sequences were submitted to GenBank (KP781884-KP781912).

## Results

The prevalence of trichuriasis among human participants was 46.2% (95%CI: 37.5-55.1%) and of 46 pigs sampled, five were egg-positive. After chemoexpulsion 697 *Trichuris* were collected from 10 humans, and 62 *Trichuris* were obtained from a pen containing four pigs.

Morphometric analysis was conducted on 49 pig worms and 64 human worms (Tables 1 and 2). There was a tendency for pig worms to be longer than human worms, with differences in total, anterior and posterior body lengths between male pig and human worms. Spicule length did not differ between male worms from pigs and humans. Discriminant analysis using posterior length, anterior length and posterior width correctly classified 100% of male worms (n = 29) with respect to host species, but only 69.3% of female worms (n = 75).

**Table 1:** **Morphometric characteristics of male** ***Trichuris*** **derived from humans and pigs in Ecuador**

**Characteristic**	**Human-derived** ***Trichuris*** **(n = 28)**		**Pig-derived** ***Trichuris*** **(n = 6)**		**P-value**
	**Median (IQR)** ^**a**^	**Min.-Max.**	**Median (IQR)**	**Min.-Max.**	
Anterior length	20 (18–21)	4-27	25.5 (23.5-27.75)	19-30	0.006
Posterior length	11 (10.25-13)	6-17	15 (13.75-16)	13-16	0.002
Total length	32 (28.25-34.75)	15-40	41.5 (38–42.5)	32-44	0.002
Anterior width	0.08 (0.07-0.10)	0.06-0.17	0.12(0.08-0.15)	0.07-0.16	0.06
Posterior width	0.48 (0.43-0.54)	0.37-0.82	0.69 (0.55-0.99)	0.55-1.00	0.0006
Spicule length	2.66 (2.56-2.73)	1.51-3.01	2.44 (2.14-2.89)	2.11-2.96	0.21

All measurements are in mm.^a^ Interquartile range.

**Table 2:** **Morphometric characteristics of female** ***Trichuris*** **derived from humans and pigs in Ecuador**

**Characteristic**	**Human-derived** ***Trichuris*** **(n = 36)**		**Pig-derived** ***Trichuris*** **(n = 43)**		**P-value**
	**Median (IQR)** ^**a**^	**Min.-Max.**	**Median (IQR)**	**Min.-Max.**	
Anterior length	20.5 (14.5-25)	5-35	25 (18–32)	2-38	0.03
Posterior length	10 (9–12)	5-17	10 (8–12)	3-22	0.28
Total length	30 (24–36.75)	14-47	33 (28–44)	11-55	0.12
Anterior width	0.09 (0.07-0.10)	0.06-0.18	0.10 (0.08-0.12)	0.05-0.17	0.18
Posterior width	0.57 (0.54-0.62)	0.49-0.83	0.69 (0.52-0.79)	0.34-1.05	0.02

All measurements are in mm.^a^ Interquartile range.

PCR-RFLP analysis was carried out on 59 pig worms (one pig pen) and 82 human worms (four hosts). All human-derived worms showed identical ITS-2 restriction patterns (bands at 340, 220 and 130 bp) and 18S patterns (360 and 170 bp). The majority of pig-derived worms demonstrated the same restriction pattern for ITS-2 (490 and 130 bp) and 18S bands at 360, 130 and 40 bp. Two pig *Trichuris* showed a “heterozygous-type” ITS-2 pattern (490, 340, 220 and 130 bp). One of these also showed a “heterozygous-type” 18S pattern. It was not possible to resolve the 18S pattern for the other sample.

**Figure 1:**
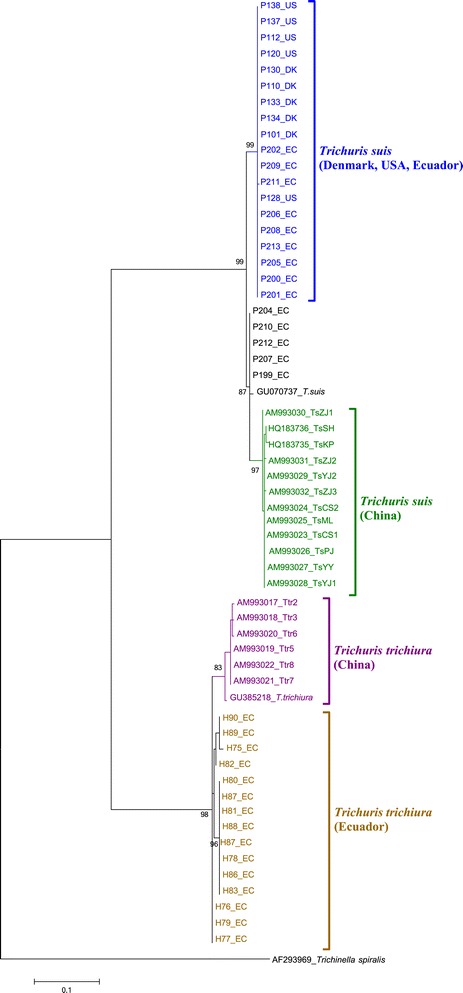
**Maximum likelihood tree based on the** ***rrn*** **L gene using Tamura-Nei with gamma distribution as the substitution model and** ***Trichinella spiralis*** **as an outgroup. Bootstrap values above 80 are reported.** *Scale bar*: number of base substitutions per site. *Sample key*: first letter indicates host (H – human, P- pig); last two letters indicate country of origin (UG – Uganda, EC – Ecuador, DK – Denmark, US – USA); numerals indicate unique worm ID. A neighbour joining tree showed a very similar topography.

Upon phylogenetic analysis of the mitochondrial *rrn*L marker, all Ecuadorian human-derived worms clustered in a distinct group but in the same clade as Chinese human-derived worms (Figure 1). Ecuadorian pig-derived worms were separated into two groups with affiliations to pig worms from Denmark and USA or to Chinese *T. suis*. The *rrn*L sequences for pig *Trichuris* which showed heterozygous-type ITS-2 and 18S patterns (P207_EC and P208_EC) clustered with Ecuadorian pig *Trichuris* sequences.

## Discussion

Here we present the first comparison of *Trichuris* worms derived from pigs and people in Latin America, specifically in rural Ecuador. We observed significant differences in certain morphometric characteristics, but there was morphological overlap between worms from different host species. We found that pig worms were longer than human worms in Ecuador, whereas human worms were longer in Uganda [3]. Consistent with the literature [15], discriminant analysis was able to correctly classify male worms, but not female worms, with respect to host origin. Given the fact that they can represent adaptations to a particular host species [16], morphometric characteristics must be interpreted with caution when used to distinguish between *Trichuris* species.

In the two PCR-RFLP analyses, all human–derived worms showed restriction patterns characteristic of worms derived from humans in other locations (ITS-2) [6] or that were expected based on *in silico* analyses of GenBank sequences (18S). Similarly, all mitochondrial *rrn*L gene sequences from human worms clustered together in a distinct clade from the Ecuadorian pig-derived worms. Thus, there was no evidence of zoonotic transmission of *Trichuris* worms from pigs to people, in contrast to Uganda [6].

Intriguingly, two out of 59 pig-derived worms showed “heterozygous-type” restriction patterns in the ITS-2 and 18S (in one case) PCR-RFLPs, the first time heterozygotes or mixed template profiles have been noted in pig *Trichuris*. This pattern may be explained by cross-infection and genetic exchange between *Trichuris* species by introgression or hybridization or through retention of ancestral polymorphisms within multi-copy ribosomal arrays that predated speciation [17]. As the *Trichuris* prevalence in humans is higher than in pigs, perhaps due to rapid expulsion of the worm population in pigs [18] or regular pig deworming (A.S., O.M.Pogoreltseva, personal communication), in this setting it may be more likely for pigs to become infected with human *Trichuris* than vice versa. Nevertheless, a more extensive analysis of *Trichuris* from pig and human hosts is necessary to confirm this and place the genetic exchange event within a likely time-frame.

The mitochondrial *rrn*L gene analysis confirmed the genetic distinction between human and pig *Trichuris* [4]. Ecuadorian and Chinese human worms were found in two groups showing phylo-geographic isolation. Some Ecuadorian pig *Trichuris* clustered with *T. suis* from Denmark and the USA, and some with Chinese *T. suis*. Pigs were originally imported into Ecuador from the Iberian Peninsula during Spanish colonization, with subsequent imports from Europe and the USA over the course of the 20th century [19]. Thus genetic similarity between *T. suis* from Ecuador and *T. suis* from Europe and USA is not surprising. The similarity of five worms to *T. suis* from China, maybe due to the introduction of European and Chinese pig breeds in the 20th century when pigs were imported from China to enhance commercial traits of European breeds [20].

## Conclusions

Molecular epidemiological studies can provide important insights into parasite transmission dynamics. Although there appears to be no zoonotic transmission of *Trichuris* in Ecuador, there is evidence of (ancestral) genetic exchange between *T. trichiura* and *T. suis*, warranting additional genetic sampling.
